# Basic Research on Whitefly Molecular Biology: A Foundation for Innovative Pest Management Strategies

**DOI:** 10.3390/cimb48060605

**Published:** 2026-06-08

**Authors:** Sonia Hussain, Georg Jander

**Affiliations:** 1 Department of Entomology, The Ohio State University, Wooster, OH 44691, USA; 2 Boyce Thompson Institute, Ithaca, NY 14853, USA

**Keywords:** whitefly, *Bemisia tabaci*, pest control, basic research, molecular biology

## Abstract

*Bemisia tabaci* (whitefly; Hemiptera: Aleyrodidae), a complex of morphologically similar but genetically distinct species, causes enormous agricultural damage worldwide. Farmers incur billions of dollars in losses each year from whiteflies, both through direct feeding damage and from the transmission of numerous plant viruses. Important crops that are heavily damaged by whiteflies include tomato, eggplant, cucumber, cotton, cucurbits, beans, and cassava. The global invasiveness and persistence of *B. tabaci* are largely attributed to its exceptional biological traits. Understanding these traits is essential for developing effective, long-term pest management strategies. This review describes in detail how the basic biology studies of *B. tabaci* provide a foundation for developing pest management strategies. Specifically, we discuss: (1) insights into the development of insecticide resistance can guide resistance management strategies; (2) knowledge of natural enemies supports the advancement of biological control approaches; and (3) understanding plant–insect interactions reveals molecular targets for innovative pest management solutions. We also examine emerging research trends and offer future perspectives on how ongoing studies may drive the development of next-generation control strategies (RNA interference, clustered regularly interspaced short palindromic repeats—CRISPR-associated protein 9 (CRISPR-Cas9), and horizontally transferred genes as targets).

## 1. Introduction

Invertebrate pests cause 30–40% of global agricultural yield losses [1,2]. Phytophagous hemipteran insects, particularly whiteflies, aphids, and mealybugs, are a major agricultural concern, primarily due to their polyphagous nature and ability to transmit numerous bacterial and viral diseases [3]. Among 1556 whitefly species belonging to 161 genera [4], *Bemisia tabaci* (Hemiptera: Aleyrodidae) is one of the world’s most economically important pests, damaging crop plants by sucking phloem sap and transmitting more than four hundred plant viruses, mostly begomoviruses [5,6,7]. *Bemisia tabaci* is a small insect, about 1 mm in length, with the haploid males being somewhat smaller than the diploid females [8]. The *B. tabaci* life cycle is 21–30 days, starting from eggs that hatch and pass through four nymphal instars before molting into adults. Each female lays about 200–300 eggs in its lifetime, allowing the rapid development of large whitefly populations [8].

**Figure 1:**
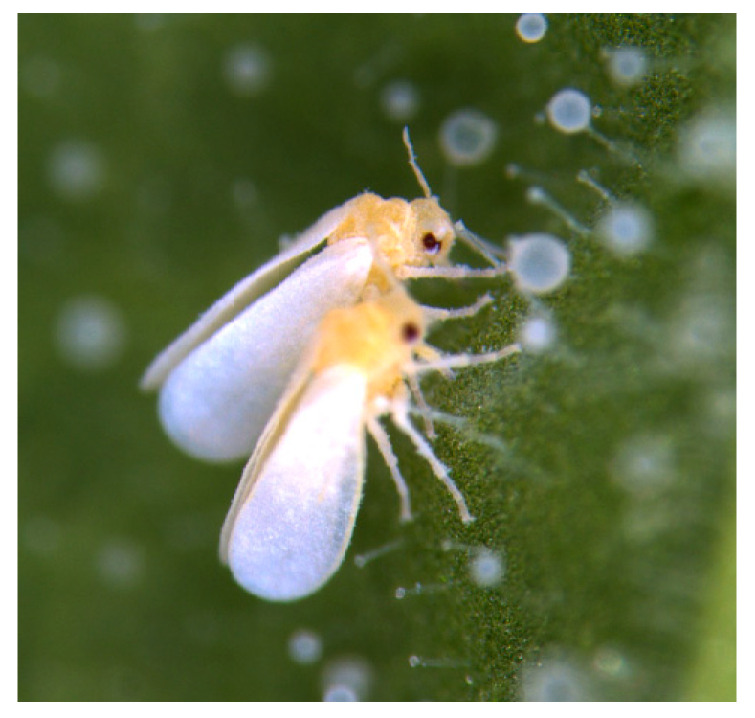
Adult *Bemisia tabaci* on a tobacco leaf. Photo credit: Honglin Feng.

*Bemisia tabaci* (Figure 1) was first discovered on tobacco plants in Greece and Florida in 1889. During 1930s, it gained notoriety as an agricultural pest for transmitting tobacco leaf curl disease in Indonesia and East Africa, as well as for transmitting cotton leaf curl disease in West Africa and Sudan. Subsequently, *B. tabaci* spread around the world, with infestations detected in the Indian sub-continent (now Pakistan) in 1929 [9], Iran and Sudan in 1950s [10], E1 Salvador in 1961 [11], Mexico in 1962 [12], Turkey in 1975 [13], Israel in 1976 [14], Thailand in 1978 [15], and Arizona and California in 1981 [16]. In many of these cases, *B. tabaci* infestations reached outbreak levels due to agricultural intensification and improper pest management. From 1960s, outbreaks of *B. tabaci*-associated viruses became more problematic in crops such as cotton, cassava, soybean, and tomato in tropical and subtropical areas. In the past three decades, most newly emerging crop plant viruses have been transmitted by *B. tabaci* [5].

Whitefly infestations and their associated viral diseases collectively cause billions of dollars in losses around the world every year. Henneberry and Faust [17] list notable outbreaks and their consequent economic losses in different regions of the world. In 1991, India suffered a 300 million United States dollar (USD) loss due to whitefly infestation of bean crops [17]. In 1993, more than half a million USD loss was estimated in the USA, and in next few years (1994–1998), cotton growers spent 154 million USD to control whiteflies [18]. From 1992 to 1997, a 5 billion USD loss was estimated in Pakistan due to cotton leaf curl disease [19]. Cassava mosaic disease and cassava brown streak disease, which are transmitted by *B. tabaci*, cause more than 1 billion USD annual losses in Africa [20]. In China, a total of 1.5 billion USD in losses was estimated from 2010 to 2012 due to whitefly-transmitted geminiviruses [21].

*Bemisia tabaci* is a species complex comprising at least 46 cryptic species (Table 1: [22]), including Africa, Sub-Saharan Africa 1–5, Asia I, Asia I-India, Asia II 1–12, Asia III, Asia IV, Asia V, Australia, Australia/Indonesia, China 1–5, Indian Ocean, Italy 1, Ru, Middle East Asia Minor I-II (MEAM), MEAM K, Mediterranean (MED), New World 1–2, Japan 1–2, and Uganda. These species are morphologically similar, but have different biological characteristics, e.g., host plant preferences, virus transmission capacity and specificity, and insecticide resistance levels. The worldwide distribution of the *B. tabaci* species complex is described in Table 1. Some of these are indigenous species and a few, notably MEAM1 and MED, are highly invasive. The molecular marker *mitochondrial cytochrome oxidase 1* (*mtCOI*) has been widely used to classify *B. tabaci* cryptic species [23,24]. The species complex can be resolved into reproductively isolated species by using a threshold of >4% genetic divergence in the *mtCOI* sequence [25]. Recently, due to the availability of whole-genome sequences [26,27], integrated approaches, including genome comparisons [28] and population genetics, are being used to delimit the *B. tabaci* species complex, while also describing the genetic mechanisms that contribute to the success of *B. tabaci* as a pest. Although some species appear to be reproductively isolated, there is also evidence for between-species hybridization of whiteflies [29,30,31].

The estimated genome (*n* = 10) size of *B. tabaci* is ~690 Mb [32]. Investigations of horizontally transferred genes in different insect species showed that *B. tabaci* is a clear champion at integrating genes from bacteria, plants, and fungi into its genome [33]. Li et al. (2022) examined 218 insect genomes and found horizontally transferred genes in 192 of them [33]. With at least 170 horizontally transferred genes, *B. tabaci* has more than any other tested insect species in this study. At least some of these horizontally acquired genes contribute to the success of *B. tabaci* as a plant pest.

An extraordinary set of biological characteristics has made *B. tabaci* a successful worldwide pest. Exploring the biological traits that contribute to *B. tabaci* survival strategies is crucial for the success of future pest control approaches. In this review, we describe how understanding the basic biology of *B. tabaci* is leading to the development of new pest management strategies, as well as presenting future perspectives related to the current basic research on *B. tabaci* and how they will lead to new pest control approaches.

## 2. Improving Control of *B. tabaci* Through Insecticide Resistance Management

### 2.1. Mechanisms of Insecticide Resistance

Since its initial emergence as an agricultural pest, chemical pesticides have been deployed extensively for *B. tabaci* control. However, *B. tabaci* also has a tremendous potential to develop insecticide resistance, making this one of the most important factors influencing the invasiveness of some *B. tabaci* species [34,35]. The MED species is the most invasive due to its unusually high propensity for insecticide resistance. In an untreated field, MEAM1, which has better survival advantages and fecundity, can outcompete the MED. However, in the presence of regular insecticide treatments, MED often replaces MEAM1, indicating a strong contribution of insecticide resistance to the invasiveness of this species [36].

Three classes of insecticides with different modes of action are commonly used to control whiteflies: (1) organophosphates and carbamates, (2) pyrethroids, and (3) neonicotinoids. Table 2 summarizes the resistance level and resistance mechanisms in the *B. tabaci* species from different locations. Here, only the most invasive species are presented. Numerous review and research articles describe the resistance status of native whitefly species [34].

Organophosphates and carbamates: Organophosphates and carbamates (OPs) are inhibitors of the acetylcholine esterase (AChE), a key enzyme in neurotransmission. They bind to the active site and inactivate the enzyme by phosphorylating or carbamylating the serine residing in the active site, leading to the desensitization of the nervous system and eventually causing the death of the insect [56]. Both MEAM1 and MED populations from different regions have developed high levels of resistance against most OPs (Table 2). Two major resistance mechanisms against OPs have been reported in *B. tabaci*: carboxylesterase (COE) mediated metabolic resistance and AChE insensitivity due to point mutations [36]. A few target site mutations in the *Ace1* gene (described in Table 2) have been associated with OPs resistance.

Pyrethroids: In the late 1980s, the use of OPs as insecticides gradually was replaced by pyrethroids [57]. Pyrethroids bind to and disrupt the function of voltage-gated sodium channel, causing hyperexcitation and in some cases nerve blockage. Two specific point mutations (Table 2) in the gene encoding the sodium channel cause insensitivity to the pyrethroids in *B. tabaci* [58]. Both MEAM1 and MED populations show varying levels of pyrethroids resistance [36].

Neonicotinoids: Neonicotinoids are the most recently developed class of insecticides. They target nicotinic acetylcholine receptors (nAChRs) and cause various symptoms, including hyperexcitation, lethargy, and paralysis. Studies have shown the development of resistance in whiteflies against even this new class of insecticides in several countries, including Brazil, China [44,53], Colombia, Egypt [41], Germany [59], Greece, India [34], Iran [60], Israel [61], Italy, Pakistan [42], Spain [62], Sudan [63], Turkey [64], and the USA [49,65,66,67]. MED populations have shown resistance to higher levels of neonicotinoids, e.g., acetamiprid, imidacloprid, and thiamethoxam, than MEAM1 populations [68]. However, there are tradeoffs associated with insecticide resistance, causing whiteflies carrying resistance genes to perform less well in the absence of insecticide selection [69,70]. Additionally, the insecticide resistance level in whiteflies varies according to the growth stage of the insects [71].

Certain endogenous *B. tabaci* genes have key functions in mediating resistance to neonicotinoids. For example, *CYP6CM1* and P-glycosyltransferase *UGT352A3* expression are correlated with resistance against imidacloprid, thiamethoxam, and acetamiprid in different Chinese *B. tabaci* populations [47,72]. However, no consistent polymorphism was observed in the promoter of the imidacloprid-resistant strain, which overexpresses *CYP6CM1*, relative to the imidacloprid-susceptible strain, indicating that the *CYP6CM1* expression level is regulated by a trans element. CREB, a basic leucine zipper transcription factor, directly regulates *CYP6CM1* expression by binding to a cAMP response element-like site in the promoter region. *CREB* is overexpressed in the imidacloprid-resistant strain, and its expression is regulated by the extracellular signal-related kinase (ERK) and p38 mitogen-activated protein kinase (MAPK) signaling pathways [73]. Thus, the *CYP6CM1* regulatory pathway provides pivotal genes that are potential targets for *B. tabaci* control. Reducing mRNA of *CYP6CM1* or other genes associated with insecticide resistance in the *B. tabaci* by RNA interference (RNAi, described in more detail below) also would be a strategy for controlling imidacloprid resistance.

### 2.2. Resistance Management

Several strategies for managing *B. tabaci* insecticide resistance have been proposed and implemented.

(1) Insecticide rotation: The risk of insecticide resistance increases when successive generations of a pest are exposed to insecticides with the same mode of action. The rotation of insecticides with different modes of action has proved to be effective in delaying resistance development in *B. tabaci* [33]. For more successful pest control, insecticides with different modes of action must be rotated after one *B. tabaci* generation (24–30 days). Along with insecticide rotation, additional parameters such as infestation level, coexistence of other pests, and pesticide resistance history should be taken into consideration.

(2) Avoiding cross-resistance: Insecticide resistance generally occurs through two major mechanisms: increased detoxification process and target site point mutations [64]. Insecticides having similar binding target sites or similar detoxifying mechanisms tend to develop cross-resistance. Cross-resistance to neonicotinoids is relatively common. Thiamethoxam and imidacloprid-resistant strains tend to show cross-resistance to other neonicotinoids but not to abamectin and pyrethroids [65,66]. However, some neonicotinoid-resistant strains show cross-resistance to other insecticides (e.g., abamectin and carbosulfan) [67]. This suggests that cross-resistance can develop in *B. tabaci* irrespective of class and mode of action of the insecticides. Knowing the resistance levels and their mechanisms in *B. tabaci* populations is important for avoiding the development of cross-resistance.

(3) Blocking the pathway of resistance: Blocking and/or knocking down the resistance genes is an important strategy for making *B. tabaci* more insecticide sensitive. RNAi-mediated expression silencing of the CYP6CM1 gene sufficiently increased imidacloprid sensitivity in an otherwise resistant strain [63]. Similarly, RNAi of a *TRPV* gene decreased the resistance to afidopyropen in *B. tabaci* [68] and RNAi of endocuticle structural glycoprotein increase susceptibility to matrine [74]. In addition to the use of RNAi technology in insecticide resistance management, there are a several other ways in which RNAi could be used to control *B. tabaci* (described in detail in subsequent sections).

Enzyme inhibitors are also used along with insecticides to increase insecticide sensitivity. Three inhibitors, piperonyl butoxide, diethyl maleate, and triphenyl phosphate, have synergistic effects with certain insecticides. AChE and GST activities are significantly inhibited by the piperonyl butoxide and diethyl maleate, respectively [67]. Piperonyl butoxide has a synergistic effect with many insecticides, including acetamiprid, afidopyropen cyantraniliprole, flupyradifurone, imidacloprid, and thiamethoxam [66,67,69,70,71]. Similarly, triphenyl phosphate shows a synergistic effect with chlorpyrifos and thiamethoxam [67,72].

(4) Regulating resistance-associated symbionts: Whiteflies contain endosymbiont bacteria that have been coevolved with their hosts over millions of years. Seven to eight endosymbionts are found in whiteflies, including the primary endosymbiont *Candidatus Portiera aleyrodidarum* (Oceanospirillales) and secondary endosymbionts in the genera *Arsenophonus* (Enterobacteriaceae), *Cardinium* (Bacteroidetes), *Hamiltonella* (Enterobacteriaceae), *Hemipteriphilus* (Proteobacteria), *Fritschea* (Chlamydiales), and *Wolbachia* (Rickettsiales) [21]. The presence of endosymbionts is associated with insecticide resistance in some *B. tabaci* populations. Further research on the mechanisms of interactions between *B. tabaci* and its endosymbionts could provide potential strategies for decreasing the insecticide resistance by regulating endosymbiont functions.

(5) Enhancing insecticide efficiency: Most pesticides are sprayed as emulsified concentrates and wettable powders that have limited absorption capacity and stability. The integration of nanotechnology provides improved pesticide application methods. Nanostructures loaded with pesticides have been used to control different pests, e.g., *Helicoverpa armigera*, *Spodoptera litura*, and *Tetranychus urticae* [73,74]. These formulations provide better stability and smart release of active agents, improved absorptive capacity, and effectiveness against insects, with limited adverse effects. Nano-biopesticides, for instance, geraniol-encapsulated nanoparticles also are effective for controlling *B. tabaci* [75,76]. Geraniol encapsulated in chitosan also is attractive for *B. tabaci*, indicating its potential use in trap devices [77].

Integration of new technologies, as well as combination of insecticides with other control methods [75], provides ways to enhance the efficacy of currently available insecticides and rationally design new insecticides. For example, understanding the chemical and functional properties of proteins interacting with insecticides can provide the basis for virtual screening of chemical libraries by designing homology models. This is also helpful for the rational design of novel insecticides that have high target specificity and low risk for non-target organisms. The draft genome of the MEAM1 species indicates some genetic novelties, including the expansion of gene families associated with insecticide resistance and detoxification [25]. So, based on this new knowledge, there may be novel ways to manage insecticide resistance in the *B. tabaci*.

## 3. Biological Control Strategies Utilizing Natural Enemies

Using natural enemies like parasitoids and predators as biological control agents is an environmentally friendly pest control strategy. Biological control methods address concerns related to the hazardous environmental impact of excessive insecticide use. Furthermore, use of natural enemies is also an attractive approach in the regions where growers and/or consumers prefer organic foods.

Altogether, 115 species have been identified as parasitoids of *B. tabaci* [76], though only a few of these have been used for agricultural pest control (Table 3). These parasitoids have different mechanisms for parasitizing *B. tabaci*. Some *Eretmocerus* species lay eggs underneath *B. tabaci* nymphs and, after hatching, they penetrate the nymphs, whereas others lay eggs directly inside of the nymphs (usually 3rd instar). *Eretmocerous californicus* directly punctures *B. tabaci* nymphs and sucks out nutrients [77]. *Eretmocerus mundus* and *Encarsia pergandiella* can locate their hosts by sensing volatile semiochemicals (kairomones) emitted from *B. tabaci*–infested foliage and honeydew [78,79,80]. After laying eggs, *E. mundus* marks the host nymphs with pheromones, enabling other females to avoid these marked hosts. This oviposition strategy increases the efficacy of the parasitoids in controlling pest populations [81].

Parasitoids that will be used as biocontrol agents are reared on plants with *B. tabaci* or other host insects for use as biocontrol agents. For mass rearing, parasitoids are released to lay eggs in *B. tabaci* nymphs feeding on plants. After 2–3 weeks, dead nymphs and/or pupae, which contain numerous parasitoids, are collected from the leaves. They are counted volumetrically and used as biocontrol agents in fields and greenhouses [79]. Both seasonal and inundative methods are used to release the parasitoids on plants.

Multiple factors must be considered when using parasitoids as biological control methods for managing whiteflies. The selection of parasitoids corresponding to a particular species of *B. tabaci* is necessary to establish efficient pest control. For instance, *E. formosa* perform better on the MEAM1 than the MED species [82]. The host species that is used for rearing the parasitoids matters for the subsequent parasitism. If they were previously reared on the same host species, *E. formosa* establish better parasitism on MEAM1 whiteflies [82]. Plant species and virus infection of the plants also contribute to the shaping of parasitism [83]. Quantitative differences of volatile compounds among different plant species and between virus-infected and healthy plants may explain this parasitoid behavior. These factors and others need to be considered before the application of parasitoids as a biological control method.

**Table 3:** Parasitoids as biocontrol agents of *B. tabaci*.

Parasitoids	Plants	Comments	Field/Greenhouse	Reference
*Encarsia hispida*	Cabbage, Cotton, Ornamental plants	Provide successful results	In field and greenhouse	[84,85]
*Encarsia formosa*	Vegetables, Ornamental plants	Need regular release	Greenhouse	[86,87]
*Encarsia sophia*	Cabbages, Green bean, Papaya, Tomato	Banker plant system for long term control	Greenhouse	[88,89,90]
*Eretmocerus eremicus*	Pepper	Weekly release	Greenhouse	[91]
*Eretmocerus hayati*	Cotton, Tomato	Periodic release	Controlled environment	[92,93]
*Eretmocerus melanoscutus*	Cabbage		Contained environment	[89,90,94]
*Encarsia lutea*	Cotton, tomato		Protected environment	[84,95]
*Encarsia bimaculata*	Cassava	Average control	Contained environment, Greenhouse	[96]
*Eretmocerus mundus*	Pepper, Sweet potato	Weekly release	Greenhouse, Protected plant growing facilities	[91,97]
*Eretmocerus emiratus*	Collard, Eggplant		Contained environment	[98]

At least 150 arthropod species have been identified as *B. tabaci* predators. Some of these have a dual role in being plant-feeding pests that also consume whiteflies [99]. However, to date, only a few of these predatory insects have been used commercially in fields and greenhouses to manage whitefly infestations. *Amblysieus swirskii*, a predatory mite, proved to be successful in controlling *B. tabaci* on sweet pepper in both greenhouses and fields [100,101]. The combined use of parasitoids and predators provided better results in controlling whiteflies, as compared to using a single natural enemy (see Table 4 for examples) [102]. The selection of the right combination of natural enemies depends on their interactions with one another and their fitness in the environment. Although the use of natural enemies as whitefly biocontrol agents can give satisfactory results, it is insufficient for high-density infestations and in unsuitable environments. Nevertheless, biological control can contribute significantly to integrated pest management practices.

## 4. Pest Management Approaches Based on Plant-Insect Interactions

### 4.1. Bemisia Tabaci-Plant Interactions

The plant immune response to phloem-feeding insect pests can be explained as a zig-zag model that has been proposed for plant-pathogen interactions [107]. Zig: As the first line of defense, plants detect herbivore-associated molecular patterns (HAMPs) through cell surface receptors (e.g., receptor-like proteins, RLPs). Receptor-like proteins transduce the signal downstream to activate HAMP-triggered immunity. Zag: Pests suppress this immunity by effector-triggered susceptibility, whereby effector molecules from the pest change the cell structure and functions by interacting with the susceptible (*S*) genes of the plants. Zig: Resistance (*R*) genes of plants recognize the effectors that lead to the hypersensitive response referred to as effector-triggered immunity. Zag: Pests keep on evolving new effectors to suppress the host immunity. These zig-zag processes of plant-insect interactions are not separate but mechanistically connected [108]. Therefore, the impact of a specific effector depends upon the presence/absence of *R* and *S* genes of the host plant. To develop effective control strategies against *B. tabaci*, it is essential to identify and understand the functions of *B. tabaci* effectors and their interactions with the *S* and *R* genes of the plants.

*Bemisia tabaci* enhances its performance by modulating host plant defenses and increasing their susceptibility. On the other hand, plants respond to whitefly feeding by inducing several phytohormone-mediated defense pathways. MEAM1 and MED repress the jasmonic acid (JA) signaling pathway and activate genes in the salicylic acid (SA) signaling pathways in both *Arabidopsis thaliana* and tomato [109,110]. Whitefly effectors, which have evolved to suppress host plant resistance, contribute to the altered regulation of JA and SA responses. Generally, effectors refer to the protein molecules (salivary proteins) secreted by the pest during feeding from the host plant. However, effectors also include any factors/molecules (e.g., from honeydew or eggs) of the pest that alter the host-cell structure and function. Salivary secretions mostly consist of proteins but also include long non-coding RNAs [111] and small RNAs [112] that influence plant responses. Not all salivary proteins/components act as effectors; some have other roles such as structural components of the salivary sheath [113]. Effectors in *B. tabaci* honeydew also can increase the SA levels of the host plants, thereby suppressing insect-targeted defenses [114].

#### 4.1.1. *Bemisia tabaci* Salivary Effectors Modulate Plant Defenses

Different bioinformatic data mining techniques have been used to identify whitefly salivary effectors. The majority of these effectors were identified based on transcriptomic data analysis, whereby transcripts encoding signal peptides without a transmembrane domain are mined for the putative effectors [115,116]. This approach is easy to use but detects a large number of putative genes, many of which may not be the actual effectors because proteins with signal peptides are not necessarily secreted into the plant but instead function internally in the whitefly. Some of the putative genes can be shortlisted based on their similarity with the other known effectors and gene expression analysis under different inducing conditions. Two protein analysis approaches have been used to identify *B. tabaci* effectors: (1) determining enzymatic activity in artificial diet, and (2) proteomic analysis of phloem exudates and plant tissue from which whiteflies have fed [117]. Proteomic analysis of plant tissue and phloem exudates is a better approach compared to transcriptomic data analysis and measuring enzymatic activity in an artificial diet. However, a limitation is that effector proteins are dispersed in host plant tissues, and the quantity of whitefly salivary proteins in the phloem and plant tissue can be very low. This approach also depends on having well-annotated genomes of both the whitefly and the host plant, so that effector proteins can be differentiated from the host proteins.

To date, several whitefly effectors have been identified. In a transcriptomic study of MED, 295 genes were predicted to encode secretory proteins that potentially function as effectors [118]. Huang et al. (2021) reported 171 salivary proteins including 34 *B. tabaci*-specific proteins through transcriptomic and proteomic analysis of the MED species [119]. *Laccase 1* (*Lac1*) was identified in the MED salivary gland transcriptome and is the first whitefly effector analyzed for its mode of action [120]. *Lac1* contains three Cu-oxidase domains, which are reported to contribute to metal ion metabolism, plant metabolite detoxification, and lignocellulose digestion. *Lac1* is expressed in the salivary gland and midgut, and expression increases when *B. tabaci* feed on tomato plants compared to artificial diet and on JA-treated plants compared to control plants. So, this suggests a role for Lac1 as an effector in modulating plant defense response. Whitefly *Lac1* is closely related to *Lac1* from other phloem feeding insects, i.e., *Acyrthosiphon pisum*, *Diaphorina citri*, *Nephotettix cincticeps*, and *Nilaparvata lugens* [120]. So, Lac1 could be considered a “core effector” because multiple pests have similar *Lac1* genes with potentially similar functions. Although Lac1 is highly abundant in *B. tabaci* eggs, as compared to the nymphal stages, its function in the eggs remains unknown [120].

There are likely to be many additional core effectors in whiteflies and other phloem-feeding insects. Huang et al. reported 97 whitefly salivary proteins that have putative orthologs in the 22 other insect species [119]. *Bemisia tabaci ferritin* (*BtFer1*) was identified in MED and shares 56–58% similarity with the ferritins of *A. pisum*, *Myzus persicae*, and *Diuraphis noxia* [117]. BtFer1 suppresses H_2_O_2_-mediated oxidative signaling, cellulose deposition, proteinase inhibitor activation, and JA-mediated signaling pathways.

Two effectors, Bsp9 and Bt56, are highly conserved orthologs identified in MEAM1 and MED [116], respectively, with only one amino acid difference in their protein sequence. They are reported to interact with two different plant targets (transcription factors). Bsp9 interacts with the WRKY33 transcription factor and prevents WRKY33 localization in the nucleus. The WRKY33 transcription factor is required for activating pathogen-responsive mitogen-activated kinases (MPK3 and MPK6). So, Bsp9-WRKY33 interaction prevents WRKY33 from activating MPK3 and MPK6, which is evidence for the effector role of Bsp9 in modulating the plant immunity [116]. Bt56 is reported to interact with a KNOTTED 1-like homeobox (KNOX) transcription factor (NTH202) in tobacco [118]. Similar to the Bsp9-WRKY33 interaction, Bt56-NTH202 interaction prevents NTH202 movement into the nucleus. In maize, KNOX1 regulates some of the genes involved in SA and JA pathways. In planta expression of Bt56 increases the production of SA but doesn’t affect the JA level, with a resulting increase in whitefly performance. However, it is not yet confirmed that the Bt56-NTH202 interaction directly influences the SA and JA levels in the plants [115].

Some *B. tabaci* salivary effector genes have been targets for knockdown with RNAi. Effector genes are attractive targets because they appear to have important functions in the interactions of whiteflies and their host plants. Genome-scale analyses of closely related *B. tabaci* species suggest that salivary effectors evolve relatively slowly [121]. This lack of sequence diversity, which indicates an essential function, makes salivary effector genes attractive as RNAi targets. Further investigation of salivary effectors and their functions will provide a better understanding of *B. tabaci*-plant relationships and may lead to better control strategies.

#### 4.1.2. Plant Defense Responses to *B. tabaci*

(1) Secondary metabolites and volatile organic compounds

**Figure 2:**
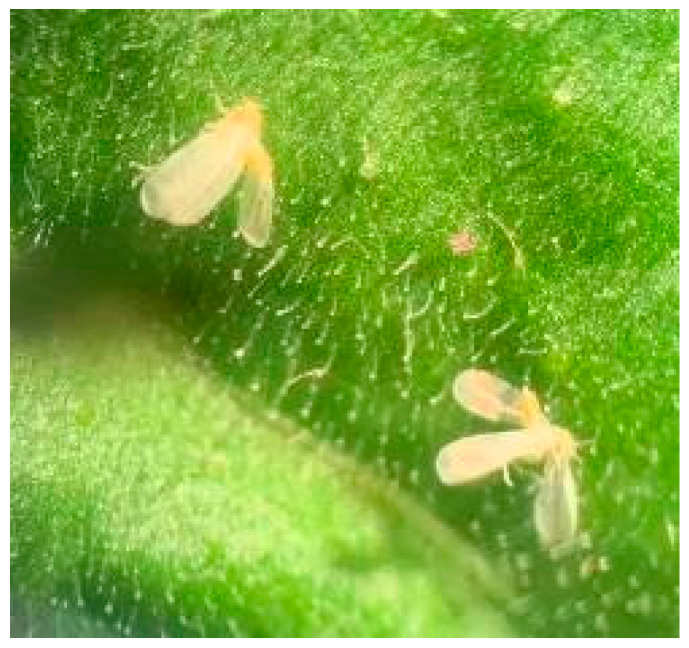
Adult *Bemisia tabaci* trapped by sticky acylsugar exudates on a *Nicotiana benthamiana* leaf. Photo credit: Honglin Feng.

Plants have developed a variety of defense mechanisms against the whitefly attack. Physically, trichome density, color, stickiness, and texture of leaves influence susceptibility to whitefly attack [122,123]. Within-species natural variation in such traits can provide whitefly resistance and is a potential plant breeding target [124]. Chemically, plants produce secondary metabolites (e.g., terpenoids, acylsugars, glucosinolates, and phenolic compounds) and emit volatile organic compounds (VOC; e.g., ocimene and myrcene) in response to whitefly attack. Whitefly infestation increased the level of several terpenoids in tobacco plants, indicating their involvement in the plant defense response against whiteflies. The positive effect on plant resistance to *B. tabaci* of one of these terpenoids, cadinene, was confirmed by the gene silencing and overexpression experiments [125]. In tomato, higher acylsugar content on the leaf surface is associated with reduced whitefly infestation [126]. Whitefly entrapment by acylsugars of *N. benthamiana* (Figure 2) was reduced by knockout of *ASAT2* (*Acylsugar Acyltransferase 2*), which also made the plants more suitable as hosts for whitefly feeding [127]. Elbaz et al. (2012) reported that high levels of aliphatic glucosinolates decreased the average oviposition rate of MEAM1 and MED species and reduced the survival of MED species on the *A. thaliana* [128]. In a similar study, Markovich et al. (2013) found that high-level accumulation of aliphatic and total glucosinolates content in *A. thaliana* reduced the oviposition preference of MEAM1 species [129]. However, Li et al. (2021) reported that the fecundity of MEAM1 and Asia II 3 species remain unaffected at the natural level of glucosinolates in *Brassica* plants [130].

Many additional plant secondary metabolites, for instance catechin, cafeic acid, chlorogenic acid, ferulic acid, p-coumaric acid, rutin, and other phenolic compounds, are involved in conferring whitefly resistance [122,131]. Flavonoids in tomato promote whitefly resistance by interfering with the function of a salivary effector [132], as well as by enhancing reactive oxygen species and callose deposition [133]. Understanding how secondary metabolites function to deter whitefly infestation provides information for future biopesticide development. Moreover, upon whitefly feeding, plants emit volatile organic compounds that can attract natural enemies. For example, whitefly infestation of tomato plants attracts the predator *Macrolophus basicornis* [134]. Zhang et al. (2013) reported that a SA-induced volatile compound β-myrcene attracts the *B. tabaci* parasitoid *E. formosa* to host plants [80]. Similarly, upon whitefly feeding, tomato plant emits β-myrcene and β-caryophyllene, which help the *E. formosa* to locate the infested plant [135]. Genetic engineering of tomato terpene synthase successfully produced plants with greater attractiveness of the parasitoid *Eretmocerus corni* [136].

(2) Plant defense proteins

As part of their effector-triggered immunity, plants produce defense proteins in response to *B. tabaci* feeding. Protease inhibitors, which inhibit proteases that are essential for digestion in the insect gut, are found in many plant species. Du et al. (2022) used silencing and overexpression experiments to show that tobacco cysteine proteinase inhibitor A-like protein NtCYS6 reduces whitefly fecundity on tobacco plants [137]. They also demonstrated the localization of NtCYS6 in the whitefly gut. In another study, van de Ven et al. (2000) reported that whitefly infestation increased the expression of a defense protein *β-glucosidase* in the squash plants [138]. Similarly, whitefly infestation increased the expression of *β-1,3-glucanase*, *chitinase*, and *peroxidase* in cassava plants [139]. Additionally, tomato and soybean infested with whitefly showed higher activity of two plant defense proteins, polyphenol oxidase and peroxidase [140,141]. The functional analysis of these and other defense proteins will provide relevant information for updating whitefly management strategies.

### 4.2. Control Strategies Based on Knowledge of B. tabaci-Plant Interactions

Using current knowledge about the changes that *B. tabaci* induce in the plants and how plants respond to *B. tabaci* infestation, various whitefly management strategies that strengthen endogenous plant defense systems can be implemented. Three strategies that have been explored in this respect include: (1) Semiochemical-based approaches, (2) Natural host plant resistance, and (3) Expression of foreign genes.

#### 4.2.1. Semiochemical Based Approaches

Semiochemicals are signals emitted by an organism to elicit responses in another organism of the same species (e.g., pheromones) or different species (e.g., allelochemicals). Whiteflies can sense the host plant’s volatile compounds through their odorant binding and chemosensory proteins [142], allowing them to discriminate between host plants, and even differentiate virus-infected and non-infected host plants [143].

A semiochemical-based management strategy exploits natural volatile signaling processes to manipulate insect behavior. For example, plant volatile profiles can be manipulated through genetic engineering to deter *B. tabaci*. 7-Epizingiberene is a sesquiterpene that is produced and stored in the glandular trichomes of some tomato species and has a repellent effect on herbivores. Bleeker et al. (2012) introduced a biosynthetic pathway for the production of 7-epizingiberene to cultivated tomato from the wild tomato, thereby increasing resistance to *B. tabaci* and several other herbivores [144]. Similarly, enhanced production of terpinolene, a volatile monoterpene, repelled *B. tabaci* adults [145]. The synthesized volatile chemicals also can be applied exogenously on the plant as a spray or with slow dispenser. Shi et al. (2016) suggested the use of SA-mediated plant volatiles to affect the plant-whitefly interactions and encouraged investigation of why the viruliferous whiteflies are less sensitive than non-viruliferous whiteflies to repellent plant volatiles [146].

As another pest control approach, semiochemicals that attract whiteflies can be used to deploy traps in the field. Some attractive chemicals are expensive to synthesize and may be chemically unstable in the field. To overcome this limitation, semiochemical analogous with better stability can be designed through chemoenzymatic synthesis [147]. For further success of such approaches, it is important to broaden the research on the olfactory system of whitefly and crop plant volatile profiles to successfully design and implement semiochemical-based approaches in the field.

#### 4.2.2. Natural Host Plant Resistance

It is often hypothesized that crop plants lost the defensive genes during domestication, making them less resistant to pests than their wild progenitors [148]. Natural resistance in the wild plants (including individual genes and quantitative trait loci (QTL)) can be utilized in breeding programs to bring back insect resistance into cultivated crops. Germplasm resources for many crops, including cotton, legumes, okra, and tomato, have been screened to identify genotypes that could potentially serve as donors of *B. tabaci* resistance for breeding programs [149,150,151,152,153]. Among these, tomato plants have been the most extensively studied for QTLs and specific genes related to whitefly resistance. Two QTLs *Wf-1* and *Wf-2,* which confer resistance to whitefly, have been identified in the tomato species *Solanum pennellii*, *Solanum galapagense*, and *Solanum habrochaites* [152,154,155]. Mata-Nicolás et al. (2021) identified a major QTL associated with the density of type IV trichomes, which confer resistance against whiteflies, in *Solanum pimpinellifolium* [156]. The *Mi-1* gene is a resistance gene that was initially found to confer resistance against root-knot nematodes but later it was also reported to impart resistance to phloem-feeding insects, including whiteflies [157]. Aslam et al. (2023) conducted functional characterization of Mi-1.2-like orthologs in cotton cultivars and described their potential involvement in whitefly resistance [158].

#### 4.2.3. Expression of Foreign Genes

Genetic engineering has been successfully used to express foreign genes, including *Bacillus thuringiensis* (*Bt*) endotoxins (Cry proteins) [159,160,161] chitinases [162], biotin binding proteins [163], alpha-amylase inhibitors [164], and protease inhibitors [165], in plants to control insect pests. Expressing Cry proteins (*Bt* endotoxins) in plants provides successful control of chewing insects and large-scale adoption of such genetically engineered crops expressing these Cry proteins reduces the use of chemical pesticides. However, transgenic crops expressing Cry proteins generally are not effective against sap-sucking pests. Nevertheless, modified versions of Cry proteins have been effective against aphids [160], *Lygus* species [161], and, more recently, whiteflies [166].

Some insecticidal proteins that confer *B. tabaci* resistance have been identified from different sources. Expressing a fungal β-glucosidase gene in tobacco provided increased resistance against *B. tabaci* [167]. In another study, fungal β-glucosidase gene expression in tobacco via chloroplast transformation resulted in enhanced whitefly and aphid resistance [168]. Similarly, transplastomic expression of *Pinellia ternata* agglutinin in tobacco and cotton plants provided broad-spectrum resistance against whiteflies, aphids, and lepidopteran pests [169,170,171]. TMA12, a protein from ferns that are intrinsically resistant to whiteflies, produced promising results when expressed in the cotton [172]. Phloem-specific expression of two insecticidal proteins, onion leaf lectin and *Hadronyche versuta* neurotoxin (Hvt), in tobacco resulted in high *B. tabaci* mortality [173]. Based on these results, developing transgenic plants conferring protein-mediated resistance against insects is likely to be a sustainable one-time investment. However, biosafety concerns need to be addressed prior to commercial cultivation of these plants.

## 5. New Biotechnological Interventions for Whitefly Research and Pest Control

### 5.1. RNA Interference

During past two decades, RNA interference (RNAi) has emerged as highly targeted and efficient tool to elucidate gene function, control pests, and prevent virus spread in plants [174]. RNAi is a post-transcriptional gene silencing process in the organisms. RNAi mechanisms involve three main steps: (1) double stranded RNA (dsRNA) corresponding to the target gene is introduced in the host cell and Dicer enzymes digest the dsRNA into short interfering RNA (siRNA, 21–25 nt long), (2) the RNA-induced silencing complex (RISC) takes up the siRNA duplex and degrades the passenger strand, and (3) RISC guided by the guide RNA locates mRNA fragment complementary to the guide RNA strand to degrade the target mRNA. The *B. tabaci* machinery of RNAi is similar to that of aphids. Upadhyay et al. (2013) first identified components of the RNAi machine including helicase, *PAZ*, *RNaseIIIa*, *RNaseIIIb*, double-stranded RNA-binding fold (DSRBF), and *Sid1* in *B. tabaci* transcriptomic data [175]. More recently, genome analysis of MED species identified 33 predicted genes potentially involved in the whitefly RNAi pathway [27,176]. Elucidating of the function of these genes will provide a better understanding of the RNAi mechanism in *B. tabaci* and improve its efficiency in whitefly management.

Broadly, two different delivery methods have been used for delivering RNAi constructs to insect pests. For host-mediated RNAi, the host plant on which the target insect feeds is used to express constructs, either transiently or as stable transgenes. As an alternate approach, the requirement for genetic engineering can be eliminated by direct application of RNAi constructs to host plants.

#### 5.1.1. Host-Mediated RNAi by Transient Expression

dsRNA can be transiently expressed in plants by either *Agrobacterium* infiltration or virus infection. These techniques are simple, quick, and useful for the rapid screening of candidate genes for functional analysis or their effectiveness as target genes for insect control. As an example, acetylcholinesterase and ecdysone receptor genes were targeted by the transient expression of dsRNA [177]. In this study, dsRNAs were transiently expressed by the infiltration of a tobacco rattle virus vector that is commonly used to initiate virus-induced gene silencing (TRV-VIGS). Whitefly feeding on the RNAi-expressing host plants caused high mortality. Other plant-mediated silencing approaches involving TRV-VIGS also have produced promising results (see Table 5 for details). However, although these approaches are suitable for rapid functional analysis of genes, they are not applicable in the field.

#### 5.1.2. RNAi Using Stable Transgenic Plants

As an alternative to transient expression, it is possible to produce transgenic plants that continuously express dsRNAs. This technique is effective and applicable for pest control in the field. Several *B. tabaci* genes targeted by the plant-mediated RNAi effectively controlled *B. tabaci* on the transgenic host plants (Table 5). Using a combination of dsRNAs targeting more than one gene provides better and more durable results for controlling insects by plant-mediated RNAi. The availability of genome sequences and computational tools makes it possible to identify and avoid non-target effects. The chances of off-target effects can also be further narrowed down by expressing dsRNA in phloem tissue to target only sap-sucking pests. Eakteiman et al. (2019) efficiently targeted a detoxification gene (*BtGSTs5*) in *B. tabaci* by phloem-mediated RNAi [178]. Whitefly non-specific ribonucleases, particularly in the gut and saliva degrade dsRNAs, which decreases the efficiency of RNAi. Luo et al. (2017) used RNAi to silence the specific nucleases along with two target genes, aquaporin and sucrase, resulting in a significant reduction in the expression level of target genes and enhanced whitefly mortality [179].

#### 5.1.3. microRNA-Mediated RNAi

In addition to dsRNA, microRNA-mediated RNAi is an effective whitefly control method. Plant host-encoded miRNAs can regulate the genes of infecting pathogens and insects [180,181]. Guo et al. (2014) showed that the expression of insect-specific artificial miRNA is efficient in controlling aphids [182]. An in-silico study revealed that *Gossypium hirsutum* encodes miRNAs that have the intrinsic ability to target and downregulate vital whitefly genes. Wamiq et al. (2018) overexpressed *gh-miRNA166b* in cotton, applying an RNAi strategy to downregulate the mitochondrial ATP synthase gene of whiteflies feeding on cotton plants [183]. Zubair et al. (2020) engineered resistance against *B. tabaci* in *Nicotiana tabacum* by targeting three vital genes (sex lethal protein, acetylcholinesterase and orcokinin) using artificial microRNA (amiRNA) [184]. The amiRNA approach has advantages over other siRNA approaches due to reduced off-target effects and less activation of the immune responses. However, it generally has a lower knockdown efficacy [185].

#### 5.1.4. Direct Delivery of RNAi Constructs

There are some non-transgenic approaches to delivering dsRNA to whiteflies, including microinjection [186], oral feeding in artificial diet, and exogenous application (e.g., spray and stem or root absorption) [187]. Table 5 lists the RNAi techniques and different delivery methods used against whiteflies. Microinjection and artificial diet methods are usually used to elucidate gene functions in the laboratory but cannot be applied to control insects in the field. By injecting dsRNA directly into specific tissues using a microprocessor-controlled instrument, microinjection avoids nucleases in the whitefly saliva and midgut. Oral feeding of dsRNA via the artificial diet is a more convenient method and causes no physical damage. Several whitefly genes (e.g., *aquaporin 1*, *alpha-glucosidase 1*, *acetylcholine receptor subunit α*, *trehalase1*, *trehalose transporter1*, *heat shock protein 70*, and *vascular ATPase*) were successfully targeted as a means of whitefly control using the oral feeding method [188,189] (See Table 5).

Exogenous applications such as spray, root absorption (soil irrigation) and stem injections are possible alternatives to genetic engineering to deliver dsRNA [190,191]. However, to date, there are only a few studies in which exogenous application has been tested for whitefly control. dsRNA applied to tomato leaves can move systemically in the plant and be taken up by mites, aphids, and whiteflies (*Trialeurodes vaporariorum*) [192]. However, the amount of dsRNA taken up by the whiteflies was very low and insufficient to produce an RNAi effect. Although dsRNA, which inhibits gene expression, can be effective as an insecticidal spray, RNA stability remains a challenge in the field. However, formulation of dsRNA with nanomaterials increases the efficiency of dsRNA treatments [193,194,195,196,197]. Similarly, modification of RNA constructs to make them more resistant to degradation has been used successfully for control of *Diaphorina citri* [198,199], and it may be possible to also apply this technology for whitefly control.

A leaf-mediated dsRNA uptake method can be used to silence the ecdysone gene in whiteflies [200]. The method involves submerging the petioles of tomato leaves in the dsRNA solution, which is then consumed by the whiteflies while feeding on the leaves. This technique has proven to be effective in silencing target genes in the whiteflies [199]. Similarly, a topical spray method was used to deliver dsRNA to whiteflies, effectively targeting the *heat shock protein 70* (*hsp70*) and *fasciclin 2* (*fas2*), which resulted in high whitefly mortality [201]. As an additional benefit, targeting *hsp70* reduced the virus transmission capacity of the whiteflies. This report proved that dsRNA can be applied as a spray to control whiteflies in the field, an approach that likely will not be hazardous as chemical insecticides.

#### 5.1.5. Potential RNAi Resistance in Whiteflies

Development of target-site resistance of whiteflies against RNAi of highly conserved essential genes may be unlikely, particularly if more than one gene is targeted at the same time. However, if RNAi is widely deployed for whitefly control in the field, it is likely that they will rapidly develop resistance by other means, such as RNA degradation or reduced uptake. In beetles that were targeted by RNAi (*Leptinotarsa decemlineata* and *Diabrotica virgifera*), resistance due to reduced uptake developed within a few generations, both in the laboratory and in the field [202,203]. Further basic research on the uptake and processing of RNA by whiteflies will be needed to help pre-empt the inevitable development of resistance to RNAi.

Genes that are unique to *B. tabaci* are good targets for RNAi because there are less likely to be negative effects on beneficial insect species. For instance, *B. tabaci* genome has some expanded gene families specifically associated with insecticide resistance (cytochrome P450s and UDP-glucuronosyltransferases), detoxification and virus transmission (cathepsins, phosphatidylethanolamine-binding proteins) compared to other hemipterans insects [26]. These expanded gene families possibly contribute to global invasiveness and could be targets for RNAi.

**Table 5:** Genes targeted in the *Bemisia tabaci* using RNA interference.

Genes	Mechanism/Function	Delivery Method	Gene Knockdown Effect Results/Comments	References
A. Effector genes				
*Lac 1*	Detoxification of secondary plant metabolites	Artificial feeding	Decreases whitefly survival on the host plant	[120]
*BtE3*	Block hypersensitive response (HR) in host plant Up-regulate SA pathways and suppress JA mediated defense	Plant mediated RNAi	Reduces phloem ingestion, reduces fecundity and survival	[204]
*BtFer1*	Because of their ability to Fe^2+^ binding and ferroxidase activity suppresses the H_2_O_2_ mediated oxidative signals in host plants	Artificial diet feeding	Enhances the induction of JA mediated defense signaling pathway, increases callose deposition, production of proteinase inhibitor, reduces whitefly survival on tomato	[117]
*Bt56*	Interacts with the KNOTTED 1-like homeobox (KNOX) transcription factor NTH202, activates SA pathway,	Artificial diet feeding device	Interrupts phloem feeding, decreases whitefly performance	[115]
B. Osmoregulatory genes				
*Aquaporin* (*AQP*)	Contributes to osmoregulation in phloem feeder by transferring dietary water across the alimentary tract epithelium	Plant mediated RNAi	Causes whitefly mortality	[194]
*Calcitonin* (*CAL*)	Transport salts and water and is involved in insect diuresis	Artificial diet and enhancement of RNAi efficiency with nanoparticles	Causes less water excretion in the whitefly	[205]
C. Sugar metabolism and transport-associated genes				
*Alpha glucosidase 1*	Transglycosylation	Artificial diet	Causes significant mortality	[188]
*Trehalase1*, and *Trehalose transporter1*	Hydrolyse and transport the trehalose			
D. Detoxification genes				
*BtGSTs5*	Hydrolyzes aliphatic- and indolic-glucosinolates, enables *B. tabaci* to feed on glucosinolate-producing plants	Plant-mediated RNAi /Phloem-mediated RNAi	Prolongs the developmental period	[178]
*CYP6DW4*	Detoxifies the insecticide dimpropyridaz	Artificial diet	Increases *B. tabaci* sensitivity to dimpropyridaz	[206]
E. Thermotolerant genes				
hsp70	Heat shock protein	Artificial diet	Increases mortality after 3 days of incubation at 25 °C	[207]
hsp23 and hsp 70	Heat shock proteins/ play role in heat tolerance	Artificial diet	Survival rate significantly decreased at 44 °C	[208]
F. Growth and developmental and reproduction genes				
Juvenile hormone esterase gene (*jhe*)	Regulatse metamorphosis and reproduction	Artificial diet	Effects on survival and reproduction of whiteflies, lower egg hatchability and incubation period	[209]
*BtCG5885*	Involved in embryogenesis	Injecting dsRNA	—	[186]
*BtGATAd*	Involved in the endodermal specification in both protostomes and deuterostomes		—	
*BtSnap*	Involved in cytokinesis		—	
*Vitellogenin receptor*, *BtA1VgR*	Oocytes and embryo development	Artificial diet	Decreased egg count, distorted egg laying pattern and increased mortality	[210]
*Syntaxin 1A*	Modulates sexual maturity and regulates neurotransmitter release by interacting with exocytic protein	Okra seedlings are treated with dsRNA (with modified pyrimidines) through root absorption	Significantly decreases survival of whiteflies fed on okra seedling treated with modified Syntaxin-dsRNA, as compared to unmodified dsRNA	[211,212]
G. Virus transmission genes				
*Cyclophilin* B (*CYCP*)	Involved in virus transmission	Artificial diet (for CLCuV), Plant-mediated (for TYLCV)	Knocking down of CYCP decrease the virus transmission (CLCuV and TYLCV)	[204,213]
*Knottin-1* (*knot-1*)	Control virus titer in whitefles	Artificial diet	Transmission efficiency of CLCuV increased 2-fold in cotton plants	

### 5.2. Exploitation of Endosymbionts

Endosymbiont bacteria can provide benefits to whiteflies by increasing their survival and invasiveness. Manipulating or targeting these endosymbionts could provide effective whitefly control strategies. Understanding the biology, evolution, and interaction between the whiteflies and their symbionts provides numerous opportunities to develop these strategies [214]. One of the most important targets could be disrupting the obligate relationship between whiteflies and their primary endosymbionts, *Portiera*. These bacteria provide the host with essential amino acids such as threonine, methionine, and tryptophan, which are deficient in phloem sap [215]. *Portiera* relies on the host for several metabolic pathways (e.g., the citrate cycle and glycolysis pathways) that are incomplete in these bacterial endosymbionts [216]. The extreme genomic and metabolic reduction in *Portiera* necessitates its relationship with the host. In one approach, the population of *Portier* and other symbionts was reduced by inducing autophagy in whiteflies [217]. It is difficult to target *Portiera* using RNAi unless an effective delivery method is employed, as it resides in specialized cells called bacteriocytes. It has been suggested that secondary endosymbionts can be used as an efficient delivery system for dsRNA directly into the bacteriocytes [214]. Symbiont-mediated RNAi has been reported in the blood-sucking bug *Rhodnius prolixus* and agricultural pests like *Frankliniella occidentalis* and *Thrips tabaci* [218,219]. Symbiont-mediated RNAi may also be employed in the whiteflies, but it will require the invitro culturing of genetically engineered endosymbionts.

One of the more important secondary endosymbionts in the whitefly is *Wolbachia*, which is localized in both the bacteriocytes and the hemolymph [220]. The primary method of transmission is maternal, but its presence in the hemolymph likely also allows horizontal transmission [221]. Comparisons of *Wolbachia*-infected and uninfected groups of the whiteflies prove that *Wolbachia* provides fitness benefits such as decreased growth time, increased survival rate, and improved adult longevity [222]. The same study also showed that *Wolbachia* offers some protection against parasitoids. *Wolbachia* make a nutritional contribution by producing important vitamins (riboflavin, flavin adenine dinucleotide, and folate) [223] and precursors of methionine and purine [216]. *Wolbachia* is reported to change (block and/or enhance) the viral transmission capacity of insects. Ant et al. (2023) reviewed the interactions between various *Wolbachia* strains and viruses in different host species [224]. Many *Wolbachia* strains cause cytoplasmic incompatibility that favors the spread of *Wolbachia* in the insect population. *Wolbachia* has been successfully used to control dengue on a large scale by using its ability to cause cytoplasmic incompatibility [225,226]. A similar strategy could be adopted to control the whitefly populations. The *Wolbachia* strains isolated from *Scleroderma guani* [227], *Drosophila melanogaster* [228], and *Corcyra cephalonica* [229] were successfully transfected into the whiteflies, causing cytoplasmic incompatibility demonstrating the potential of this strategy to control whitefly populations.

RNA suppression technologies other than RNAi, for instance synthetic nucleic acid-like oligomers that regulate gene expression, have been developed to control pathogenic bacteria. Sandoval-Mojica et al. (2020) used an antisense oligonucleotide, peptide-conjugated phosphorodiamidate (PPMO), to target the *Diaphorina citri* endosymbiont ‘*Wolbachia*’ and the pathogen ‘*Candidatus* Liberibacter asiaticus,’ which is transmitted by it and causes citrus disease huanglongbing [199]. PPMO is an antisense oligonucleotide composed of six-sided morpholino rings linked together and a cell-penetrating peptide that increases the bacterial uptake of PPMO. These can be designed to target the bacterial-specific mRNA. After binding to complementary mRNA, it recruits RNase which cleaves the mRNA. The one PPMO molecule can be used repeatedly, thus providing more efficiency than RNAi. Similarly, another antisense oligonucleotide (2′-deoxy-2′-floro-D-arabinonucleic acid; FANA) successfully suppresses an essential gene of *Wolbachia* in *D. citri* [230]. Because of its structure, FANA is less prone to enzymatic degradation and enhanced binding capacity to mRNA. It required a lower dose because one molecule of FANA can be used to degrade multiple copies of mRNA, thus providing enhanced efficiency. In a similar approach, these strategies can also be used to target *Wolbachia* in *B. tabaci*.

Endosymbionts are also associated with mediating whitefly-plant interactions by manipulating the plant defense system. Su et al. (2015) reported that the *Hamiltonella defensa*-infected whiteflies suppressed the JA and JA-related anti-herbivore induced defense pathways in tomato plants [231]. Investigating endosymbiont genes specifically involved in suppressing the JA pathways can provide an important clue to developing whitefly control strategies. Similarly, another study reported that *Rickettsia belli*, after transmission from whiteflies to plants suppresses, the JA pathway and triggers the SA-related gene expression, which facilitates transmission and promotes whitefly survival but make plants more resistant to fungal and viral pathogens [232]. Thus, eliminating these beneficial endosymbionts can hinder whitefly survival and invasiveness. Various studies have used antibiotics to eliminate or disrupt endosymbionts in whiteflies, reporting a negative impact on whitefly fitness [233,234]. Although applying this strategy in the field may be challenging, the use of antibiotics is an effective approach for investigating the essential functions of whitefly-endosymbiont interactions in the laboratory.

### 5.3. CRISPR-Cas in Whiteflies

The CRISPR-Cas (clustered regularly interspaced short palindromic repeats—CRISPR-associated) system has been used in different insects to knock out important genes that promote fitness or pesticide resistance. Using CRISPR-Cas to knock out cytochrome P450 genes resulted in a reduced survival rate of *H. armigera* after insecticide exposure [235]. Similarly, knocking out of a developmental gene *abd-A* in *S. litura* [236] and *Plutella xylostella* [237] led to reduced survival. CRISPR-Cas9 (CRISPR-associated protein 9) was used to knock out the olfactory receptor coreceptor gene in *S. litura*, which is involved in detecting sex pheromones [238]. This genetic alteration resulted in the inability of *Spodoptera littoralis* to sense semiochemicals. Usually, for gene editing with CRISPR in insects, the Cas9 ribonuclease complex is injected into the embryo, which is difficult for insects with small-sized eggs like *B. tabaci*. Continuous modifications and improvements in the CRISPR-Cas system addressed the limitations of injecting into small eggs. Heu et al. (2020) developed a ‘Receptor-Mediated Ovary Transduction of Cargo’ method in which they fused the Cas9 protein with an ovary-targeting peptide ligand that carried the Cas9 ribonuclease complex to the ovary, leading to the genome editing in the *B. tabaci* offspring [239]. De Rouck et al. (2024) enhanced the delivery of the Cas9 ribonuclease complex in difficult-to-transform insects, such as spider mites and thrips, using a method called SYNCAS [240]. In this method, a nanoparticle (Branched amphiphilic peptide capsules) and an endosome escape reagent (saponin) are formulated to provide a synergistic effect to increase the efficacy of the CRISPR/Cas9 system. Mocchetti et al. (2025) successfully used this method to edit *B. tabaci* with 39% efficiency [241].

The development of CRISPR-based gene drive technology in insects represents a significant advancement that brings CRISPR technology to practical application in the field. CRISPR based gene drive have been used to suppress the population in flies and mosquitoes by targeting the female viability and male sterility genes, which is called the precision-guided sterile insect technique (pgSIT). However, the effectiveness of this approach is not determined in insects with haplodiploid genome systems such as *B. tabaci*.

### 5.4. Horizontally Transferred Genes

*Bemisia tabaci* acquired certain genes from other organisms (bacteria, fungi, and plants) as horizontally transferred genes (HTGs) during the evolutionary process, which increases their adaptive power. Gilbert and Maumas (2022) identified a total of 49 whitefly genes acquired from plants in 24 horizontal gene transfer events [242]. Whiteflies use most of these genes to interact with plants or respond to pathogens. The phenolic glycoside malonyl transferase genes (*BtPMaT1* and *BtPMaT2*) were acquired by *B. tabaci* from the plants through a horizontal gene transfer event. The *BtPMaT1* gene encodes an enzyme that catalyzes the transfer of a malonyl group to a secondary metabolite phenolic glycoside, which *B. tabaci* uses to counteract plant defenses [243]. Transferring the malonyl group to phenolic glycosides renders them harmless to the *B. tabaci*. In this way, *B. tabaci* used the plant-derived genes against the plants. Similarly, a plant-derived HTG encodes “Ricin-like” protein, which is predicted to play a role in defense against pathogens. The fungal-derived HTGs in *B. tabaci* are mostly associated with lipid metabolism, carbohydrate processing and most importantly, pro-oxidant functions. Twenty of these genes function as aromatic peroxygenases (APOs) in *B. tabaci*. These fungal-derived APOs are thought to play a role in insecticide resistance and the detoxification of xenobiotic compounds [26].

Several studies have reported successful targeting of HTGs by RNAi. Xia et al. (2021) used a plant-derived gene, *phenolic glucoside malonyltransferase* (*BtPMaT1*), as a plant-mediated RNAi target and successfully conferred whitefly resistance to tomatoes [243]. Feng et al. (2023) targeted HTGs in whiteflies by virus-induced gene silencing and achieved promising results [244]. Gong et al. (2025) silenced expression of a globulin seed storage protein gene that was horizontally transferred from plants [245]. Liu et al. (2025) used RNA interference to inhibit expression of a plant-derived Δ7-sterol C5-desaturase-like gene [246]. Targeting HTGs with RNA interference has fewer chances of off-target effects because these genes are usually genus or even species-specific. Beyond their application in pest control strategies, studying the HTGs in *B. tabaci* could provide valuable insights into how the incorporation of these genes contributes to its biological adaptation and invasiveness.

## 6. Conclusions

This review highlights the translational potential of molecular discoveries for practical pest management. Extensive research into whitefly physiology and their interactions with host plants has been instrumental in developing innovative and effective control strategies. These include overcoming insecticide resistance, improving the targeted use of insecticides, and disrupting critical biological pathways in whiteflies. Key advances involve the use of RNA interference and CRISPR/Cas technologies to target essential genes, such as those involved in host plant interactions, endosymbiont functions, and horizontally transferred genes. Other promising approaches include enhancing or engineering natural plant defenses and manipulating endosymbiont communities to impair whitefly survival and reproduction. Further advances in molecular biology research of whitefly on different molecular levels will provide valuable insights that can assist scientists in designing more effective management strategies.

## Figures and Tables

**Table 1:** Worldwide distribution of *B. tabaci* species.

Region	*B. tabaci* Species
South Asia	Asia 1, Asia II 1, Asia II 5, Asia II 7, Asia II 8, Asia II 11, Asia II 13, China 3, MEAM1, MEAM K,
South East Asia	Asia I, Asia II 5, Asia II 7, Asia II 12, Australia/Indonesia, China 2, MED
East Asia	Asia I, Asia II 1, Asia II 2, Asia II 3, Asia II 4, Asia II 6, Asia II 7, Asia II 9, Asia II 10, Asia III Asia IV China 1, China 2, China 3, China 4, Japan 1, Japan 2, MEAM1, MEAM2, MED
Middle East	Asia I, Asia II 1, MEAM1, MED
Africa	Indian Ocean, New World, MEAM1, MEAM2, MED, Spain 1, Sub Saharan Africa 1, Sub Saharan Africa 2, Sub Saharan Africa 3, Sub Saharan Africa 4, Sub Saharan Africa 5, Uganda
Europe	Indian Ocean, Italy 1, Italy 2, MEAM1, MED, New World 1, Ru, Sub Saharan Africa 2, Sub Saharan Africa 3,
Australia	Australia, MEAM1
South America	MEAM1, MED, New World 1, New World 2,
North America	MEAM1, MED, New World 1
Central America	MEAM1, MED, New World 1

Data in Table 1 are from Kanakala and Murad, 2019 [22].

**Table 2:** Insecticide resistance levels and reported insecticide resistance mechanisms in the *B. tabaci* species complex (mostly invasive species) from different regions.

Insecticide	Species (MEAM1/MED)/Region/Resistance Level	Genes Contributing to Resistance (Mechanism of Resistance)	Mutations	References
Organophosphates and Carbamates				
Acephate	MEAM1 (Israel) high resistance Asia 1 (India) high resistance	Acetylcholinesterase *ace1* carboxylesterase genes (*coe1* and *coe2*)	F331W (*ace1*) Phe392Trp (*ace1*) Phe331Trp (*ace1*)	[35,37,38,39,40,41,42,43]
Carbosulfan	MED (China) Susceptible to very low NA (Egypt) moderate to high resistance			
Chlorpyrifos	MED (China) high resistance NA (Brazil) high resistance			
Malathion	NA (Pakistan) low resistance			
Methamidophos	NA (Pakistan) high resistance			
Profenofos	NA (Pakistan) low to moderate resistance			
Pyrethroids				
Bifenthrin	MEAM1 (China) moderate to high resistance MED (China) High resistance MEAM1 (USA) low	Para-type voltage-gated sodium channel	L925I and T929V (para-type voltage gated sodium channel)	[43,44,45,46]
Cypermethrin	MED (China) moderate to high MEAM1 (China) moderate to high NA (Pakistan) high resistance (USA) Moderate			
Neonicotinoids				
Acetamiprid	MED (China) moderate to very high MED (Israel) low to high NA (Brazil) high resistance MED (Germany) high resistance MED (Italy) high	Cytochrome P450 gene (*CYP6CM1* and *CYP4C64*); glutathione-S-transferase gene (*GSTd7*); nicotinic acetylcholine receptor β1 subunit gene (*Btβ1*); ATP-binding cassette subfamily G member 3 gene (*ABCG3*)		[35,37,44,47,48,49,50,51,52,53,54,55]
Dinotefuran	Asia 1 (India) low to high MEAM1 (China) low MED (China) low			
Flupyradifurone	SSA1 (Tanzania) low MED (Greece) low MED (China) low to high			
Imidacloprid	MED (China) moderate to high MEAM1 (China) moderate to high MED (Israel) Moderate MEAM1 (USA) High resistance			
Nitenpyram	MED (China) low to high MEAM1 (China) Low			
Thiamethoxam	MED (China) moderate to high MEAM1 (China) moderate to high MEAM1 (Turkey) low to high			
Other Insecticides				
Abamacetin	MED (China) no to low resistance MEAM1(China) no resistance	Cytochrome P450 gene and glutathione-S-transferase gene (*GST*)		[53]
Cyantraniliprole	MED (China) low to moderate			
Pymetrozine	MED (China) no to low resistance			

NA: the specific whitefly species was not identified in these studies.

**Table 4:** Combined use of parasitoids and predators to manage whiteflies.

Parasitoid	Predator	Plant	Reference
*Er. mundus*	*Macrolophus caliginosus*	Tomato	[103]
*Er. mundus*	*A. swirskii*	Eggplant, cucumber, sweet pepper	[101]
*Eretmocerus mundus*	*Macrolophus melanotoma*	Egg plant	[104]
*Eretmocerus eremicus*	*Geocoris punctipes*	Tomato	[105]
*Eretmocerus eremicus*	*Orius albidipennis*		[106]

## Data Availability

No new data were created or analyzed in this study. Data sharing is not applicable to this article.

## Abbreviations

The following abbreviations are used in this manuscript:

AChE	acetylcholine esterase
APO	aromatic peroxygenase
Cas9	CRISPR associated protein 9
CRISPR	clustered regularly interspaced short palindromic repeats
dsRNA	double-stranded RNA
ERK	extracellular signal-related kinase
HAMPs	herbivore-associated molecular patterns
HTG	horizontally transferred gene
HR	hypersensitive response
JA	jasmonic acid
MEAM	Middle East Asia Minor
MED	Mediterranean
MAPK	mitogen-activated protein kinase
RNAi	RNA interference
nACh	nicotinic acetylcholine receptors
OPs	Organophosphates and carbamates
pgSIT	precision-guided sterile insect technique
QTL	quantitative trait locus
SA	salicylic acid (SA)
siRNA	short interfering RNA
TRV-VIGS	tobacco rattle virus—virus-induced gene silencing
USD	United States dollar
VOC	volatile organic compounds
